# Evaluation of Vascular Structures of Living Donor Kidneys by Multislice Computed Tomography Angiography before Transplant Surgery: Is Arterial Phase Sufficient for Determination of Both Arteries and Veins?

**DOI:** 10.5334/jbsr.1719

**Published:** 2019-04-04

**Authors:** Mehmet Ali Ikidag, Erdal Uysal

**Affiliations:** 1 Department of Radiology, SANKO University Medical School, Gaziantep, TR; 2 Transplant Surgery, SANKO University Medical School, Gaziantep, TR

**Keywords:** renal artery, renal vein, CTA, nephrectomy, end stage renal disease

## Abstract

**Objectives:**

The aim of our study was to determine the efficacy of preoperative early arterial Computed tomography angiography (CTA) in donor nephrectomy, to assess the renal arterial and venous structures of donor kidneys.

**Materials and Methods:**

Seventy living donor candidates were included to this study, who had CTA for the assessment of their renal vessels in our hospital between January 2011 and January 2015. Only early arterial phase images were obtained to avoid exposing the patients from high dose of radiation. Scans were reported by two radiologists independently. The number of renal arteries, veins and their tributaries were documented. The donor kidneys were removed by two consultant surgeons, and after back-table perfusion the same details were recorded and taken as the reference findings for the operation side.

**Results:**

A total of 70 potential live kidney donors underwent renal CTA, among them fifty five patients had donor nephrectomy. A total of 140 kidneys were evaluated by CTA and the vessels of 55 harvested kidneys were compared with CTA findings. There were 40 kidneys that had at least one accessory or polar artery. There were 5 early branching renal arteries, two retroaortic and two circumaortic renal veins. Three kidneys had multiple renal veins. Operation findings were totally consistent with CTA findings in patients who underwent donor nephrectomy.

**Conclusion:**

Arterial phase CTA is sufficient for evaluation of both arterial and venous vessels of kidneys, and precontrast, venous or late phase imaging should be preserved only for chosen circumstances to avoid high radiation exposure.

## Introduction

Renal transplantation is the most efficient treatment method in end-stage renal disease. Living donor transplant surgeries are more successful than cadaver transplant surgeries due to sufficient preparation period in elective circumstances and short ischemia durations, also survival of living donor grafts are found to be longer than cadaver ones [1]. Living donor renal transplantations are being performed increasingly as their success rates are higher and there are not enough numbers of cadaver grafts [1]. Transplant surgeon’s preoperative precise information about donor’s renal vasculature is crucial for a successful graft nephrectomy, to reduce the risk of vascular injury and therefore to shorten the ischemia duration [2]. With the development of noninvasive radiological modalities, conventional angiography is rarely needed for preoperative assessment of donor kidney vessels. Computed tomography angiography (CTA) has become the first choice among noninvasive radiological modalities for the evaluation of donor’s renal vessels. It can also reveal the concomitant pathological conditions of kidneys and other abdominal structures at the same investigation [1,3]. However, many institutions perform multiphasic renal CTA to assess arteries and veins including precontrast, arterial, venous, and late-phase studies; therefore, patients are exposed to high amount of radiation. There are few studies that focus on the sufficiency of arterial phase alone. The aim of our study is to determine the efficacy of preoperative single-phase early arterial CTA in donor nephrectomy to assess the renal vascular variations, anomalies, and parenchymal pathologies of donor kidneys.

## Materials and Methods

Data about transplant donor candidates who underwent CTA for the assessment of renal vessels in *name* University Hospital between January 2011 and January 2015 were included, retrospectively. The study was approved by the Ethical Committee of *name* University and all candidates had signed an informed consent before the CT examinations. All images were obtained by the same 64-row CT scanner (Siemens Somatom Definition, Siemens Healthcare, Erlangen, Germany). Since iodinated intravenous (IV) contrast administration is mandatory for CTA examinations, patients were questioned if they had allergy history to iodinated contrast material or penicillin. Oral contrast material was not used in any of the patients. An 18 or 20 G intravenous catheter was inserted into an antecubital vein. IV contrast was delivered via a double pump injector unit (Medrad Stellant, Warrendale, Pennsylvania, USA). After they were taken in the gantry in supine position, an anteroposterior scout image was taken to determine the boundaries of imaging area. Two centimeters above the right diaphragm and below the iliac crests were marked and the CT screening area was determined as between these marks. A region of interest (ROI) was placed within the proximal abdominal aorta, above the celiac truncus level. After the beginning of contrast delivery via automated pump injector, the abdominal aorta was screened via bolus tracking software and the CTA protocol was started automatically as soon as contrast enhancement in the ROI reached 150 HU.

Iomeprol 400 mg/ml (Iomeron) or iodixanol 320 mg/ml (Visipaque) were used with four ml/sec injection rate and 2 ml/kg dose (maximum 120 ml). Forty mL of IV saline solution was delivered following IV contrast media. The parameters of CTA protocol were as follows: 64 × 0.6 mm collimation, 0.5 second rotation time, 100–120 kV and 150–450 milliamperes (mA) current in X ray tube, 0.6 mm detector thickness and reconstruction interval. Scan duration was between 8 and 11 seconds in craniocaudal direction, completed in one breath hold time in all patients. Only arterial phase images were obtained to avoid exposing the patients to high doses of radiation, as all patients had abdominal ultrasonography examination to assess kidney morphology and scintigraphy to assess functions before the CTA examination.

Source images were transferred to a separate workstation (Leonardo Workstation, Siemens Healthcare, Erlangen, Germany). In addition to axial source images, maximum intensity projection (MIP), multiplanar reformat (MPR) and three-dimensional volume rendering technique (VRT) were used to evaluate kidney vessels.

Scans were reported by two radiologists independently. Each of them had 10 years of experience in abdominal CT and CTA. The mean duration between the interpretations was approximately seven days. The number of renal arteries, veins and their tributaries were documented. Accessory and polar vessels, variations such as early bifurcation and high origins were evaluated. An artery segment originating from aorta and coursing towards renal hilum was noted as an accessory artery, while it was recorded as polar artery if it coursed towards superior or inferior pole of the kidney [4].

The donor kidneys were removed by two consultant surgeons, and after back-table perfusion the same details were recorded and taken as the reference findings for the operation side.

SPSS 13.0 software program was used for statistical analysis and p < 0.05 was considered as significant.

## Results

Seventy living donor candidates were evaluated.

**Figure 1:**
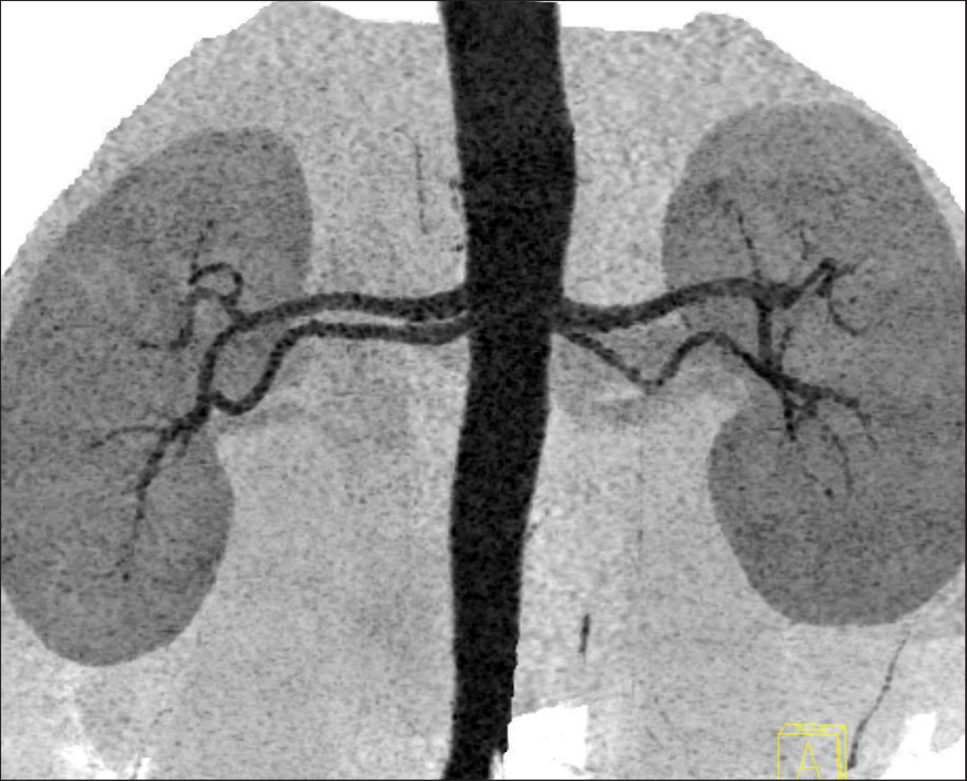
Thick slab coronal inverted gray scala MIP image depicts bilateral double renal arteries, originating close to each other from the aorta and coursing almost parallel to each other towards renal hilum.

**Figure 2:**
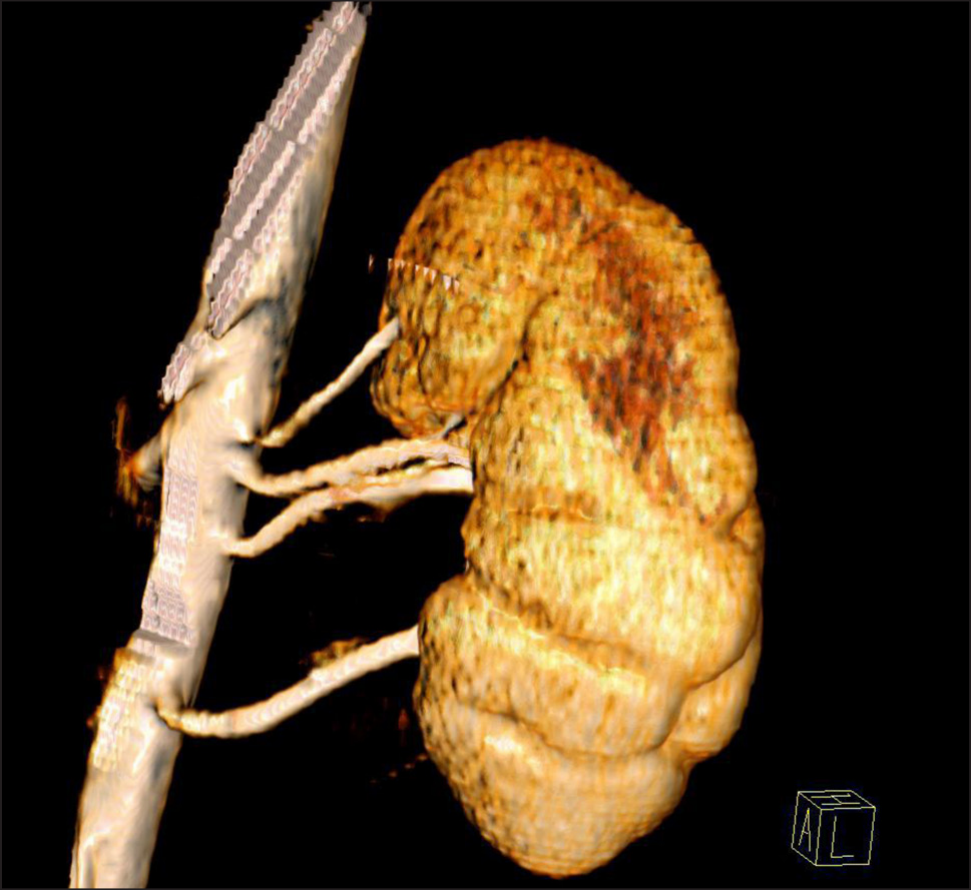
3D VRT image of left kidney. There are four arterial segments originating separately from aorta, two of them courses towards renal hilum, one to upper pole, and one to lower pole of the left kidney.

**Figure 3:**
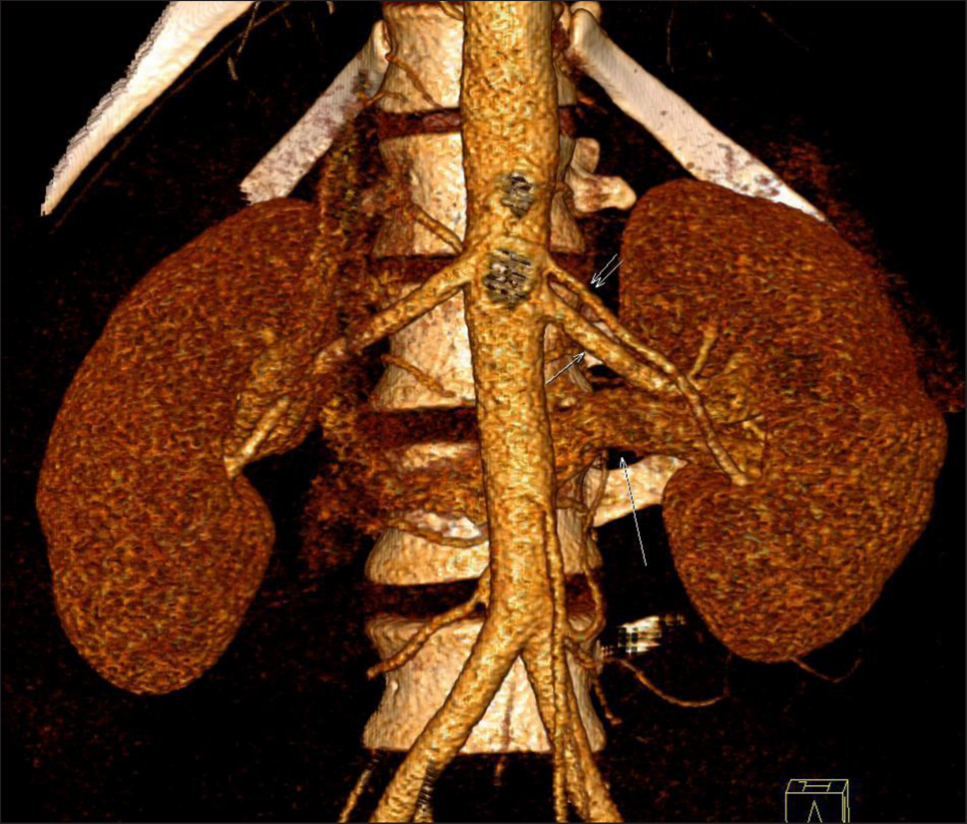
3D VRT image of both kidneys, anterior view shows left accessory renal artery (double short arrows) that originates superior to main renal artery (short arrow) from the aorta and courses parallel to it to the renal hilum. Note retroaortic left renal vein (long arrow) is clearly visible on this arterial phase image that courses behind the aorta.

**Figure 4:**
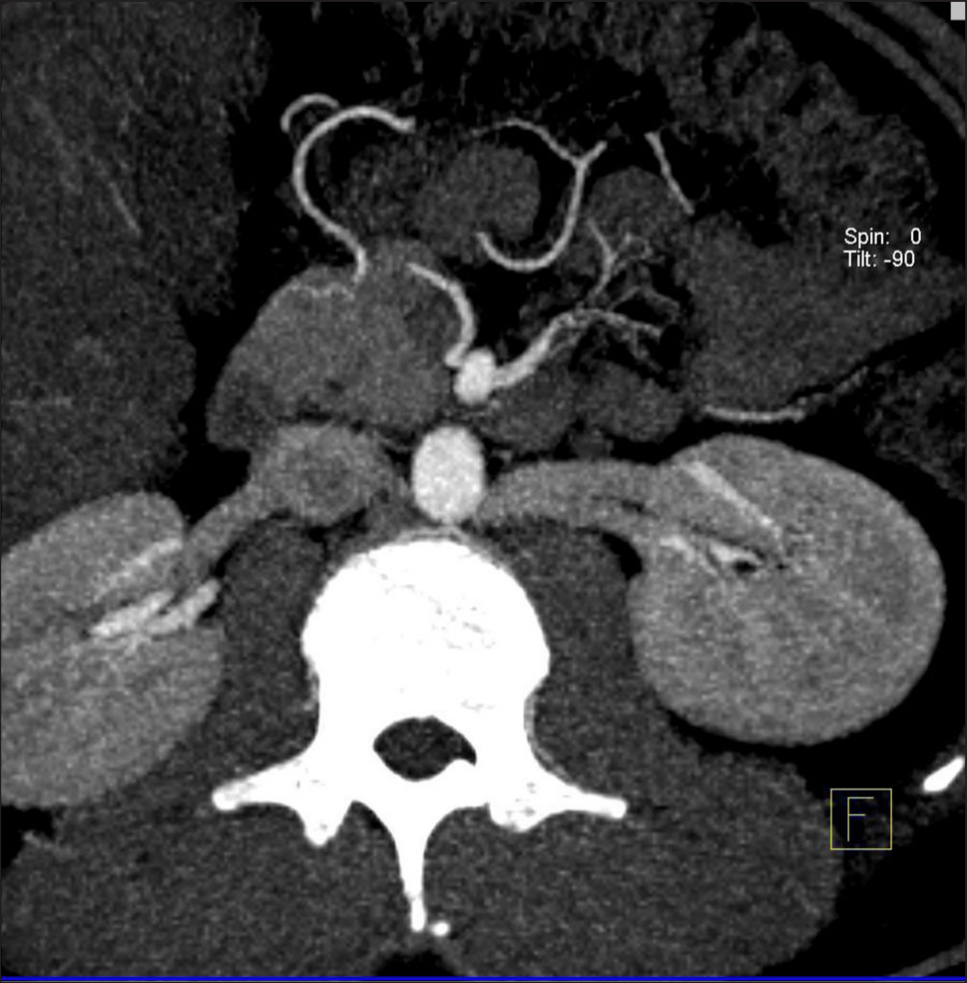
Axial MIP image shows double retroaortic left renal veins.

**Figure 5:**
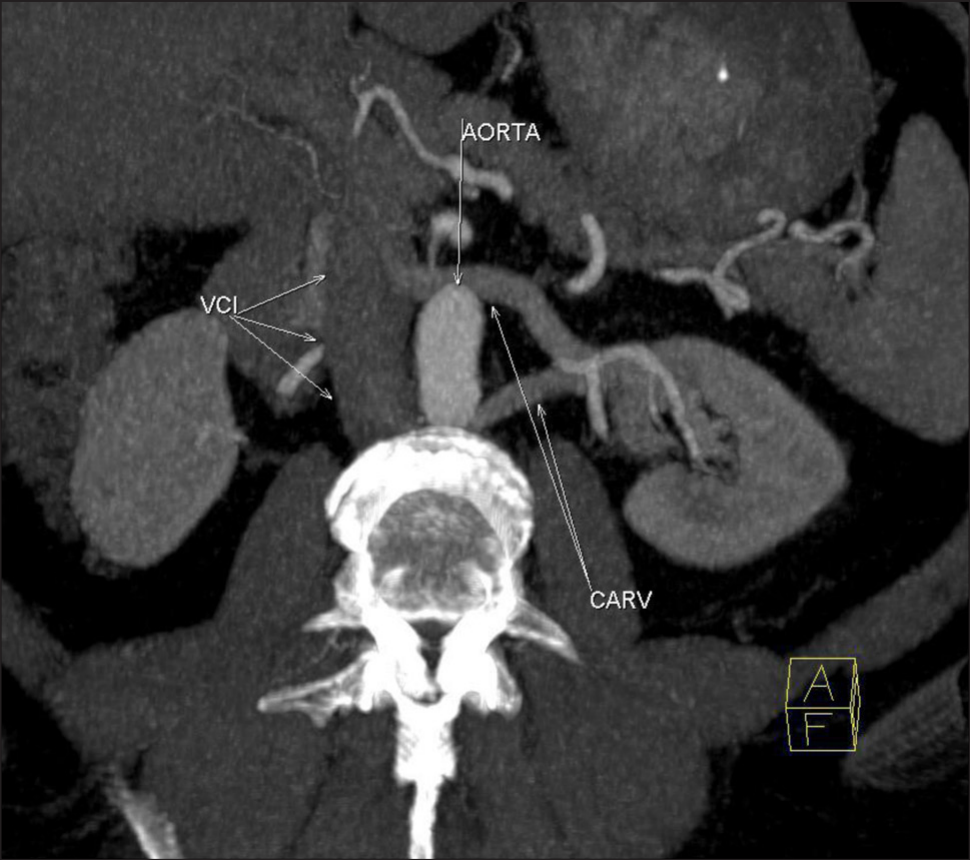
Oblique axial MIP image in arteriel phase clearly shows circumaortic left renal vein (CARV) that distal sides drain seperately to vena cava inferior (VCI).

**Figure 6:**
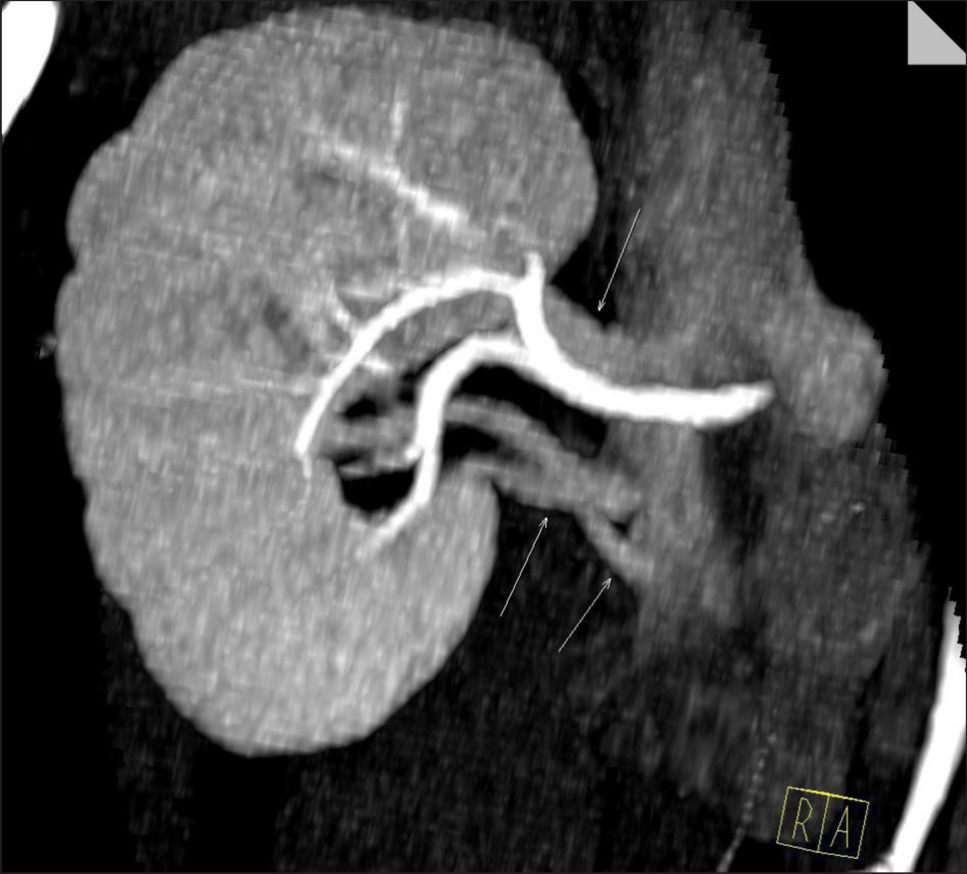
Coronal MIP image, obtained in arterial phase clearly depict 3 right renal veins that drain to vena cava separately, one courses just above the right renal artery and two others below it.

Forty two of the patients were male, and 28 were female. Mean age of donors was 46.3 ± 12.9 years. Repetition of the CTA was not needed in any of the patients. Among the 70 potential live kidney donors, fifty five had donor nephrectomy, 32 for the right kidney and 23 for the left one. Fifteen candidates were excluded from the operation, five according to CTA findings and another 10 for other reasons, such as positive serum cross-match and resignation of candidates. A total of 140 kidneys and their vessels were evaluated by CTA (Table 1), and the vessels of 55 harvested kidneys were compared with CTA findings. According to CTA findings, 100 (71.4%) of these kidneys had single renal artery. There were 40 kidneys (29.6%) that had at least one accessory or polar artery (Figure 1). One patient had 4 polar arteries instead of left main renal artery (Figure 2), and right nephrectomy was performed to prevent prolonged graft ischemia due to long duration of left nephrectomy. There were two retroaortic (Figures 3 and 4), two circumaortic (Figure 5), and three multiple (Figure 6) renal veins. One patient had double renal arteries on each side and accompanying double retroaortic renal veins on the left. Also, one patient had two renal arteries on the left that were equal in size and two polar arteries on superior and inferior each, and this patient also had three renal veins on the right that were separately draining into vena cava inferior. These two patients did not undergo donor nephrectomy due to these findings, as the recipient candidates had other relatives who had normal one renal artery and vein on each side on their renal CTA examinations.

**Table 1:** Renal arterial and venous variations encountered in 140 kidneys of renal donor candidates (n = 70).

	Right	Left	Female/Male

**Accessory renal artery**	4	3	4/3
**Polar renal artery**	9	10	8/11
**Early branching renal artery**	2	3	3/2
**High originating renal artery**	3	1	2/2
**Anterolateral originating renal artery**	3	0	1/2
**Atherosclerosis**	2	1	0/2
**Retroaortic renal vein**	0	2	1/1
**Multiple renal veins**	2	1	2/1
**Circumaortic renal vein**	0	2	1/1

There were five early branching renal arteries, three in the left side and two in the right side. One right renal artery originated prominently higher, between the celiac and superior mesenteric arteries. Three others had high origins, at the level of the superior mesenteric artery. In three patients right renal arteries were originating from the anterolateral side of aorta, more anterior than usual. One patient had calcified plaque in proximal right renal artery that did not cause significant stenosis. One patient had atherosclerotic plaques in both renal arteries that caused severe stenosis and he was excluded from operation.

Interobserver agreement was excellent. Both radiologists found the same results except two cases. One high originated right renal artery was considered as normal by one radiologist. In another case one of the radiologists reported a single right renal vein, but both radiologists agreed in double veins that were separately draining to vena cava when they revisited the images together. Operation findings were totally consistent with CTA findings in patients who underwent donor nephrectomy. Apart from vascular structures, there were cortical simple cysts in four kidneys, five adrenal incidentalomas, and one lymphocele originating behind left kidney and descending to iliac bifurcation. Lymphocele was removed during left donor nephrectomy. None of the donors had congenital malformation regarding the urinary system, except one who had renal malrotation in both kidneys. One of the donor candidates had a small calculus in the left kidney that was not apparent in ultrasound or X-ray, who underwent unenhanced urinary CT examination next day and diagnosis was confirmed, and was excluded from the operation because urinary stones are contraindicated for renal transplant donation. There was no significant difference between males and females or right and left kidneys with regards to accessory renal arteries (p > 0.05).

## Discussion

Renal transplantation is the most efficient treatment method in the end stage renal disease. Transplantation of kidneys that have one renal artery is technically easier than kidneys that have more than one renal artery. Moreover, in transplantation of kidneys with one renal artery, post-surgical rates of complication and kidney loss are lower when compared to transplantation of kidneys with more than one renal artery [5,6]. Surgeon’s preoperative precise information about donor’s renal vasculature is crucial for a successful graft nephrectomy, to reduce the risk of vascular injury and to shorten the ischemia duration and renal CTA is the most used modality for evaluation of donor’s renal vessels. If both kidneys are normal, the kidney with less complicated vascular anatomy is removed. The left kidney is preferred for laparoscopic living donor nephrectomy because it has a longer renal vein and it is technically easier to remove [7]. In some circumstances, such as complex left vessel anatomy or multiple accessory arteries, right donor nephrectomy is preferred. For that reason, CTA has an important role in choosing the appropriate donor kidney according to vasculature. Previous studies revealed that CTA has an accuracy between 95–100% in determining the donor vessels [8,9]. In our study, all CTA findings were consistent with operation findings in the harvested kidneys. There were five patients that right donor nephrectomy was preferred and five patients were excluded from the operation according to CTA findings.

Anatomic variations of the renal arteries are common in general population with different frequencies among several ethnic and racial groups [10]. Accessory renal arteries constitute the most common and clinically important renal arterial variations and can be seen in up to one-third of the normal population [11], but the probability of having more than one renal artery in both kidneys of a kidney donor is found to be low [4]. The findings in our study are consistent with previous reports.

Neymark et al. reported that a calcified plaque does not allow a vessel to close properly when clamped and may cause laceration of the intima of both the renal artery and the aorta, which may lead to life-threatening bleeding [12]. In our study, one patient had atherosclerotic plaques in both renal arteries that caused severe stenosis and he was excluded from donation.

One of the donor candidates had a small stone in the left kidney that was not apparent in ultrasound or X-ray. He underwent unenhanced urinary CT examination next day and the diagnosis was confirmed. As nephrolithiasis has been considered an absolute contraindication to live kidney donation [13,14], he was excluded from the operation.

In our study we revealed that arterial phase images are sufficient for evaluation of both arterial and venous vessels of kidneys in transplant donor candidates. Kawamoto et al. suggested that venous phase imaging is not necessary for the evaluation of renal vein anatomy in their study that included 100 potential living renal donors, since they found enough contrast enhancement in the renal veins that was sufficient to evaluate the CT images [15]. Also in our center, we obtained only arterial phase images in renal CTA to avoid exposing the patients to high doses of radiation, as all patients had abdominal ultrasonography examination to assess kidney morphology and scintigraphy to assess functions. In our center all renal donor candidates underwent routine abdominal ultrasonography examination. As ultrasonography can reveal the size, shape, localization of kidneys, also renal mass lesions, stones or calyceal dilatations can clearly be identified. Even if it is not apparent in ultrasonography, small renal stones can be easily noticed in arterial phase CTA because contrast media does not fulfill the collective system and cover the high density of a stone, just as in our case. We also did not use venous or late phases, as renal veins have enough contrast to evaluate them in arterial phase, and functions of kidneys were also evaluated in our nuclear medicine department by scintigraphy.

A recent study that focus on preoperative MR Angiography in donor candidates reveal that it is possible to determine the renal arterial and venous anatomy with high accuracy when compared with CTA, though CTA was found more reliable in the depiction of small renal veins [16]. Also MRI has the capability to evaluate pre and post-transplant renal function, parenchymal volume, renal blood flow and glomerular filtration rate [17,18,19]. The major advantages of MRI are lack of ionizing radiation and low risk of contrast reactions. Though these studies are promising, we believe that single arterial phase renal CTA would be beneficial in donor candidates who cannot undergo MRI examinations for different reasons or MRI is not achievable.

Renal vessel variations are not contraindications for transplant surgery, but having knowledge about the presence of these vessels and their courses will prevent the possible injuries or bleedings and prolonged ischemia of graft. Since all accessory or polar arteries must be anastomosed and that leads to prolonged graft ischemia and surgery duration, surgeons have tendency to transplant a kidney that has normal vessels, if possible. In our study, three kidneys were harvested despite the presence of accessory or polar arteries due to scintigraphy findings, since the better functioning kidney has to be left for the donor.

## Conclusion

Renal CTA is an important noninvasive diagnostic tool for determination of vessels of living renal donor candidates. It helps with surgery planning, choosing operation side and exclusion of donors in some cases. Arterial phase images are sufficient for evaluation of both arterial and venous vessels of kidneys, and precontrast, venous or late phase imaging should be preserved only for chosen circumstances to avoid radiation exposure.
